# Equilibrium and Kinetics Studies of Metal Ions Biosorption on Alginate Extracted from Marine Red Algae Biomass (*Callithamnion corymbosum* sp.)

**DOI:** 10.3390/polym12091888

**Published:** 2020-08-21

**Authors:** Alina Roxana Lucaci, Dumitru Bulgariu, Iftikhar Ahmad, Laura Bulgariu

**Affiliations:** 1 Department of Environmental Engineering and Management, “Cristofor Simionescu” Faculty of Chemical Engineering and Environmental Protection, Gheorghe Asachi Technical University of Iași, 700050 Iaşi, Romania; alina-roxana.lucaci@tuiasi.ro; 2 Department of Geology, Faculty of Geography, “Al.I.Cuza” University of Iaşi, 700506 Iaşi, Romania; dbulgariu@yahoo.com; 3 Romanian Academy, Filial of Iaşi, Branch of Geography, 700506 Iaşi, Romania; 4 Department of Environmental Sciences, COMSATS University Islamabad, Vehari Campus, Vehari 61100, Pakistan; iftikharahmad@ciitvehari.edu.pk

**Keywords:** alginate, low-cost biosorbent, biosorption/desorption, metal ions, wastewater treatment

## Abstract

Biosorption is a viable alternative that can be used to remove heavy metal ions from aqueous effluents, as long as the biosorbent used is cost-effective and efficient. To highlight this aspect in this study, alginate extracted from marine red algae biomass (*Callithamnion corymbosum* sp.) was used as biosorbent for the removal of Cu(II), Co(II) and Zn(II) ions from aqueous media. Biosorption studies were performed in a batch system, and the biosorptive performances of the alginate were examined as function of initial solution pH, biosorbent dosage, contact time, initial metal ions concentration and temperature. The optimal experimental conditions were found: initial solution pH of 4.4, a biosorbent dose of 2.0 g/L and a temperature of 22 °C, when over 88% of Cu(II), 76% of Co(II) and 81% of Zn(II) are removed by biosorption. The modeling of the obtained experimental data show that the Langmuir isotherm model and pseudo-second kinetic model well describe the biosorption processes of studied metal ions. The maximum biosorption capacity (q_max_, mg/g) increases in the order: Cu(II) (64.52 mg/g) > Zn(II) (37.04 mg/g) > Co(II) (18.79 mg/g), while the minimum time required to reach the equilibrium is 60 min. Moreover, the regeneration efficiency of alginate is higher than 97% when a 10^−1^ N HNO_3_ solution is used as desorption agent for the recovery of Cu(II), Co(II) and Zn(II) ions. All these characteristics demonstrate that the alginate extracted from marine algae has promising applications in the decontamination of industrial effluent containing metal ions.

## 1. Introduction

The industrial development of the recent decades has caused the contamination of many water sources with different types of pollutants around the world. Discharges of industrial effluents containing large amounts of various hazardous pollutants directly into water sources, or their improper treatment before discharge, are still considered to be two of the most important sources of environmental pollution [1,2]. Therefore, the efficient treatment of industrial effluents before discharge must be carefully controlled and monitored, in order to reduce as much as possible the negative effects of industrial activities on the environment. Particular attention should be paid to industrial effluents containing metal ions, as these pollutants are not biodegradable and have the potential to accumulate in ecosystems and human bodies [3,4]. Copper, cobalt and zinc are metal ions that are commonly found in the effluents which result from various industrial activities (such as electroplating, metal coating, paints, fertilizers manufacturing, etc.) [5,6]. These metal ions can easily react with biological molecules causing changes in the structure of proteins, nucleic acids and enzymes, with severe consequences for human health [7]. Therefore, it is important to remove such metal ions from industrial effluents before they are discharged into environment. On the other hand, the recovery of metal ions from industrial effluents and their reintroduction into the technological circuit is another aspect that must be considered in the context of the circular economy. Under these conditions, the selection of a treatment method for industrial effluents must be made taking into account both its efficiency and operating costs, as well as the possibility of recovering the removed metal ions after their treatment as easily as possible.

Among the numerous methods that can be used for metal ion removal from aqueous effluents (such as chemical precipitation, membrane separation, ions exchange, adsorption, etc.) [5,6,7,8,9], biosorption has several unique advantages, which are very important from a practical point of view. Thus, the possibility of using a large number of solid materials of biologic origin, which are cheap and readily available in most regions of the world, as well as having flexibility and adaptability to various experimental conditions [10,11,12], are just two advantages that highlight the importance of biosorption in treatment of aqueous effluents containing metal ions.

Marine algae biomass is one of the solid materials of biological origin that has been most often used to remove metal ions from aqueous media. Thus, regardless of their nature (brown, red or green algae) or the class to which they belong (micro- or macro algae), numerous studies in the literature [13,14,15] have examined the behavior of these biosorbents in the processes of metal ion retention from aqueous media, to highlight their applicative potential. This widespread use of marine algae biomass as a biosorbent in experimental studies is mainly determined by the following advantages: (i) they are easily accessible materials almost anywhere in the world, (ii) their preparation requires a small number of simple mechanical operations, (iii) most of the functional groups in their structure are of the carboxyl and hydroxyl type, which have a high affinity for metal ions from aqueous solution, and (iv) at least so far, they have quite a few other practical uses [16]. Due to these significant advantages, the retention of almost all metal ions (whether they have toxic potential or not) on marine algae biomass has been examined, and the obtained results have indicated that the efficiency of the biosorption process depends both on the nature of the metal ions adsorbed from the aqueous solution, and on the type of seaweed biomass used as biosorbent. In addition, the studies performed by Febrianto et al., 2009 [17], have shown that for a given metal ion, the brown marine algae biomass is always more efficient in biosorption process, followed by the red marine algae biomass and then by the green marine algae biomass. The higher efficiency of brown and red marine algae biomass in removing metal ions from aqueous media is mainly determined by their high alginate content from their biomass composition, compared with the green marine algae biomass [17,18].

However, the use of brown and red marine algae biomass as biosorbents in the treatment of aqueous effluents also has an important disadvantage, namely secondary pollution [19]. Secondary pollution is caused by the release into the aqueous media of color pigments and other organic compounds from the structure of the algae biomass, during the biosorption process. The release of such organic compounds during the treatment processes determines an increase in the loading with organic compounds, therefore an increase in the oxidability index of the treated effluents. This is not desirable, because it can directly affect the quality of treated water, which after the biosorption process, has a significantly low content of metal ions (removed by biosorption), but a much higher content of organic compounds [20].

Under these conditions, the extraction of alginate from marine algae biomass could be a viable alternative to eliminate secondary pollution, as this compound is mainly responsible for the high biosorptive performances of marine algae biomass [21,22]. In addition, if the extraction method is facile, the obtained alginate biosorbent can be included in the category of low-cost materials and will allow a better valorization of this kind of biomass, in wastewater treatment systems. The extraction method for alginate in alkaline media [23] meets all the conditions to be used in the preparation of this biosorbent, because it is simple and rapid, requires only a few operations, and leads the attainment of alginate in the form of calcium salt, which is suitable for the biosorption processes of metal ions.

In the Black Sea (which is the most important Romanian source of marine algae), the most common are red and green marine algae. As the alginate content of green marine algae biomass is very low, the raw material that can be used for alginate extraction is red algae biomass. This is the reason why the marine red algae biomass (*Callithamnion corymbosum* sp.) was selected in the experimental procedures.

In this study, alginate extracted from marine red algae biomass (*Callithamnion corymbosum* sp.) by an adapted alkaline method, was used as biosorbent for the removal of Cu(II), Co(II) and Zn(II) ions from aqueous media. The selection of these metal ions was made taking into account the importance and frequency of their use in industrial activities, as well as their consequences on human health. The biosorptive performance of obtained alginate was studied in detail as a function of initial solution pH, biosorbent dose, contact time, initial concentration of metal ions and temperature, in batch systems. Special attention was paid to the isotherm and kinetic modeling of the experimental data, in order to highlight the biosorption mechanism. Furthermore, the regeneration efficiency of alginate was examined to provide a complete description of the possible use of this biosorbent in the decontamination of industrial effluent containing metal ions.

## 2. Experimental

### 2.1. Reagents and Chemicals

All chemical reagents used for the experimental studies were of analytical grade. In order to prepare the stock solutions (10^−2^ mol/L) of metal ions (Cu(II), Co(II) and Zn(II)) and exactly weighed amounts of metal nitrate salts (purchased from Chemical Company, Iaşi, Romania) were dissolved in 500 mL of distilled water. After 24 h of standby, the stock solutions were used to prepare the working solutions by dilution with distilled water. HNO_3_ solution, with different concentrations, was used to adjust the initial working solution pH (measured with a pH/ion-meter MM-743 type, equipped with a combined glass electrode) and as a desorption agent.

### 2.2. Biosorbent Preparation and Characterization

Red marine algae (*Callithamnion corymbosum* sp.) were collected from the Black Sea coast, in August 2016. After washing (several times with distilled water), drying (70 °C, in air for 8 h), crushing and sieving (until a granulation of 1.0–1.5 mm), the algae biomass was used as biosorbent and for the extraction of alginate in basic media. Briefly, 5 g red marine algae biomass was treated with 100 mL of 1M NaOH solution for 4 h for the dissolution of sodium alginate from the marine algae composition. The obtained liquid phase was heated for 24 h at 50 °C and then cooled to 10 °C, using an ice bath. The cold solution was treated with 100 mL of 1M CaCl_2_ solution and vigorously stirred for 2 h, to precipitate the alginate salt. The obtained precipitate of calcium alginate was filtered, washed with distilled water and dried in air. More technical details related to the extraction procedure and the structural characteristics of the obtained alginate, were presented in a previous study [24].

Changes in the structure of alginate before and after the biosorption of metal ions were demonstrated by FTIR spectra and SEM images. FTIR spectra were recorded using a FTIR Bio-Rad Spectrometer under the following conditions: 400–4000 cm^−1^ spectral domain, resolution of 4 cm^−1^, 32 scans, KBr pellet technique, and were used to highlight the differences that appeared in the structure of the functional groups of alginate due to the biosorption processes, while the morphology of samples was examined using scanning electron microscopy (SEM microscope, SEM-Hitach S3000N).

### 2.3. Biosorption Experiments

Batch biosorption experiments were performed in 100 mL conical flasks, stirring (with 150 rpm) 25 mL of metal ions solution (Cu(II), Co(II) and Zn(II)) with a known amount of alginate, under well defined experimental conditions. The pH-dependence was examined between 2.0 and 6.5 intervals. The variation range for the biosorbent dose was from 2.0 g/L to 20 g/L. Kinetics studies were conducted at a constant concentration of metal ions (25–27 mg/L), a constant pH (4.4) and biosorbent dose (2.0 g/L), at various time intervals between 5 and 180 min. Isotherm studies were performed at three temperatures (7, 23 and 45 °C) and different initial metal ion concentrations (between 12 and 180 mg M(II)/L). The residual metal ion concentration in the solutions obtained after filtration was analyzed by atomic absorption spectrometry (AAS NovAA 400P Spectrometer, air-acetylene flame). The following equations were used for the calculation of biosorption capacity (*q*, mg/g) and removal percent (R,%):

(1) $$q\;=\;\frac{(c_{0}-c)\cdot V}{m}$$

(2) $$R\;=\;\frac{c_{0}-c}{c_{0}}\cdot 100$$

where: *c*_0_ and *c* are the initial and equilibrium metal ion concentrations in the solution (mg/L); *V* is a measure of solution (L), and *m* is the mass of alginate used in the experiments (g).

In desorption experiments, 0.5 g of alginate loaded with well-known amount of each metal ion (Cu(II), Co(II) and Zn(II)) was treated with 10 mL of 10^−1^ N HNO_3_ solution, for 3 h, after which the mixtures were filtered and the concentration of metal ions in the solution was analyzed as described above. After desorption, the alginate sample was filtered, washed with distilled water, treated with 10 mL of 10^−1^ M CaCl_2_ solution (for conditioning), and used in another biosorption cycle. Three biosorption/desorption cycles were performed for each studied metal ion. The efficiency of metal ions desorption (% Desorption) from alginate samples was evaluated basis on the following equation:

(3) $$\%\;\mathrm{Desorption}\;=\;\frac{c_{d}}{q\cdot m}\cdot 100$$

where: *c_d_* is the concentration of metal ions desorbed from alginate samples (mg/L); *q* is the biosorption capacity (mg/g); and *m* is the amount of alginate used in desorption experiments (g).

All experiments were carried out in duplicate and in all cases the standard deviation obtained from ANOVA statistical analysis were lower than 1.5%.

## 3. Results and Discussion

The extraction of alginate from marine red algae biomass (*Callithamnion corymbosum* sp.) is a simple procedure, which can be easy adapted to concrete experimental conditions. The obtained alginate, in Ca-form, is easy to separate, wash and dry [24]. This means that the obtained alginate can be included in the category of low-cost materials, and its use as a biosorbent is thus economically justified. Therefore, the biosorptive performances of this polymer should be also tested to highlight its potential applications in the environmental remediation processes. To achieve this, it is necessary to establish the optimal conditions for retaining metal ions, and to perform isotherm and kinetic modelling. For this purpose, three metal ions were selected for the experiments, namely: Cu(II), Co(II) and Zn(II), taking into account their industrial importance and their chemical characteristics.

### 3.1. Optimization of Experimental Parameters

Three experimental parameters must be taken into account in optimizing the experimental conditions for the biosorption processes: initial solution pH, biosorbent dosage and temperature. This is because initial solution pH affects the speciation of metal ions in aqueous solution and the dissociation state of superficial functional groups of biosorbent [25], the biosorbent dosage determines the number of active functional groups for the biosorption process [26], while the temperature is mainly related to the cost of the biosorption processes. Therefore, the influence of these three parameters must be examined over a wide range of variations in order to determine their optimal value.

**Figure 1:**
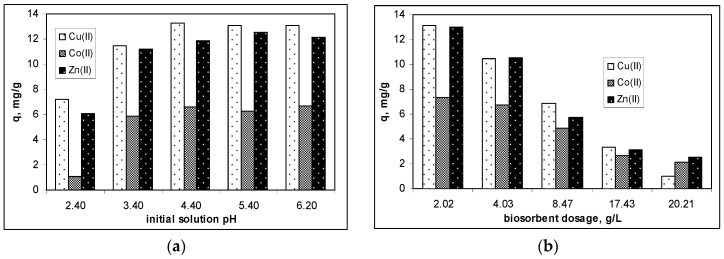
Influence of initial solution pH (**a**) and biosorbent dosage (**b**) on the biosorption efficiency of Cu(II), Co(II) and Zn(II) ions on alginate. (Experimental conditions: Co = 25.41 mg Cu(II)/L, 23.57 mg Co(II)/L, 27.10 mg Zn(II)/L; temperature = 22 °C).

Figure 1a illustrates the biosorption profile of Cu(II), Co(II) and Zn(II) ions on alginate as a function of initial solution pH. As can be seen from this figure, the lowest values of biosorption efficiency (30%) were obtained in strong acid media (pH < 3.0), but increasing the initial solution pH determined the increases in efficiency of metal ion biosorption on alginate. This increase was more pronounced in the pH range between 3.4 and 4.4, after which the biosorption efficiency remained almost constant, even if the initial solution pH increased by almost two units (Figure 1a).

This variation of the biosorption efficiency of Cu(II), Co(II) and Zn(II) ions on alginate can be explained by taking into account the elementary ion-exchange equilibriums that take place at the surface of biosorbent. In strong acid media (pH < 3.0), when the concentration of protons is higher than the concentration of metal ions from aqueous solution, most of superficial functional groups of alginate become un-dissociated, due to the ion-exchange equilibrium between Ca(II) ions and protons (see Equation (4)), and cannot interact with positively-charged metal ions from aqueous solution.

(Alg-O)_2_Ca + 2H^+^ = 2 Alg-OH + Ca^2+^ (4)

where: (Alg-O)_2_Ca is alginate in Ca-form, obtained after extraction; Alg-OH is alginate in free form.

When the initial solution pH increases, the proton concentration from the aqueous solution becomes lower than the concentration of metal ions, and the ion-exchange equilibrium that takes place at the interface is:

(Alg-O)_2_Ca + M^2+^ = (Alg-O^−^)_2_ M^2+^ + Ca^2+^ (5)

where: M^2+^ is Cu(II), Co(II) or Zn(II) ions.

Therefore, the pronounced increase in biosorption efficiency in the pH range between 3.4 and 4.4 was mainly due to the increase in the degree of dissociation of superficial functional groups of the biosorbent (Equation (4)), while the approximately constant values of biosorption capacities at pH values higher than 4.4 were determined by the fact that protons do not intervene in any way in the processes of retention of metal ions on alginate (Equation (5)). This behaviour is an advantage, in terms of wastewater treatment, because the retention of metal ions from industrial effluents using alginate as a biosorbent does not require rigorous control of their initial pH.

However, on the basis of the experimental data presented in Figure 1a, an initial solution pH of 4.4 was selected as the optimal value for the biosorption of Cu(II), Co(II) and Zn(II) ions from aqueous media on alginate. Under these experimental conditions, the values of removal percent were higher than 85% (98.52% for Cu(II) ions, 86.56% for Co(II) ions, and 86.78% for Zn(II) respectively), and the biosorption processes can be considered quantitative.

The influence of biosorbent dosage on the biosorption efficiency of Cu(II), Co(II) and Zn(II) ions on alginate was also examined in detail, and the obtained experimental results are illustrated in Figure 1b. In these experiments, the biosorbent dosage was varied from 2.0 to 20.0 g/L, while the initial solution pH was maintained at the optimal value (pH = 4.4). A significant decrease in the biosorption capacity of alginate for all metal ions was observed as the dose of biosorbent increased. The most affected by the increasing of biosorbent dosage (from 2.0 to 20.0 g/L) was the biosorption of Cu(II) ions, for which a decrease in biosorption capacity from 13.11 to 1.03 mg/g was observed, followed by Zn(II) ions (with a decrease in the biosorption capacity from 13.02 to 2.52 mg/g), and Co(II) ions (with a decrease in the biosorption capacity from 7.36 to 2.15 mg/g) (Figure 1b).

This is a typical variation, which was reported by many studies from the literature for the biosorption of metal ions from aqueous solution, and can be explained by the formation of aggregates at higher biosorbent dosage, which decrease the effective surface area for biosorption [27,28]. On the other hand, at high values of biosorbent dosage, the alginate particles are close to each other, so that the Ca(II) ions released by a certain functional group (according with Equation (5)), can be retained by an another functional group of biosorbent. This makes the number of available functional groups to interact with metal ions from aqueous solution decrease, and therefore, the efficiency of the biosorption processes will also decrease. Under these conditions, a biosorbent dosage of 2.0 g/L was considered optimal for the biosorption of Cu(II), Co(II) and Zn(II) ions on alginate, because at this value: (i) the biosorption capacities had the highest values (see Figure 1b), and (ii) the removal of studied metal ions was still quantitative, since the removal percentages were higher than 76% (88.25% for Cu(II) ions, 76.81% for Co(II) ions and 81.35% for Zn(II) ions, respectively).

**Figure 2:**
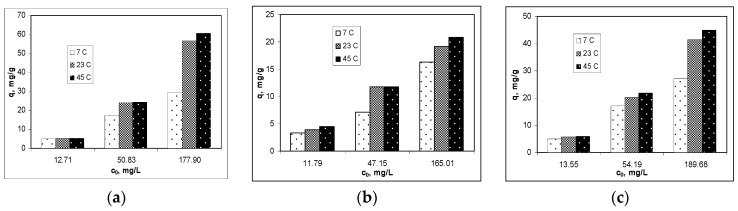
Influence of temperature on the biosorption efficiency of: (**a**) Cu(II), (**b**) Co(II) and (**c**) Zn(II) ions on alginate.

The effect of temperature on the biosorption of Cu(II), Co(II) and Zn(II) ions on alginate was examined at three different values (7°, 23° and 45 °C), and the variation of the biosorption capacities is shown in Figure 2. An increase in biosorption capacity with increasing temperature was observed for all the studied metal ions, indicating the endothermic nature of the biosorption processes. Thus for the highest metal ions concentration, the increase of temperature from 7° to 23 °C, determined an increase in the biosorption capacity of 69.90% for Cu(II), 27.69% for Co(II) and 52.20% for Zn(II) ions, respectively.

When the temperature increased from 23 °C to 45 °C, the biosorption capacities also increased, but this increase was smaller (7.06% for Cu(II), 8.66% for Co(II) and 8.67% for Zn(II) ions, respectively). Under these conditions, the ambient temperature (23 °C) can be considered optimal for the biosorption of Cu(II), Co(II) and Zn(II) ions on alginate, both in terms of the efficiency of biosorption processes and for economic reasons.

### 3.2. Effect of Contact Time and Kinetic Modelling

**Figure 3:**
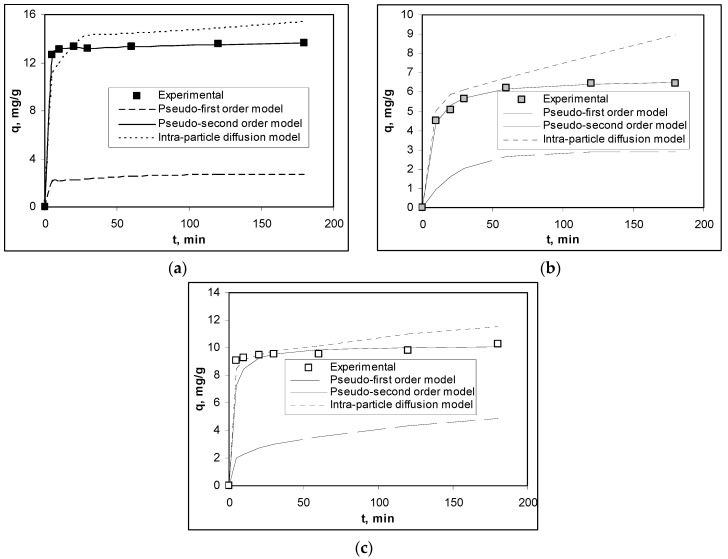
Experimental and theoretical kinetic curves obtained for the biosorption of (**a**) Cu(II) ions, (**b**) Co(II) ions and (**c**) Zn(II) ions on alginate.

The biosorption rate of Cu(II), Co(II) and Zn(II) ions on alginate was performed by measuring the residual concentration of each metal ion in solution, after separating the phases at different time intervals. The experimental variation of the biosorption capacities as a function of contact time, under optimal experimental conditions (pH = 4.4; biosorbent dosage = 2.0 g/L) is presented in Figure 3.

(Experimental conditions: pH = 4.4; biosorbent dosage = 2.0 g/L; *c*_0_ = 25.41 mg Cu(II)/L, 23.57 mg Co(II)/L, 27.10 mg Zn(II)/L; temperature = 22 °C).

More than 60% of the initial amount of each metal ion was retained on the alginate in the first 10 min (84.28% for Cu(II) ions, 63.37% for Co(II) ions and 73.71% for Zn(II) ions, respectively). This suggests that, initially, on the surface of the alginate there was a large number of functional groups available to interact with the metal ions in the aqueous solution. Therefore, the extraction of alginate from marine red algae biomass in basic media, allows the attainment of a biosorbent with high reactivity. After this time interval, the biosorption processes became slower, and reached the plateau after a maximum of 60 min. At this point, the alginate surface can be considered saturated, and insignificant variation of the biosorption capacities (1.91% for Cu(II) ions, 4.03% for Co(II) ions and 3.23% for Zn(II) ions, respectively) obtained until the end of the experiments (180 min) was due only to secondary processes of rearrangement or diffusion of metal ions inside the pores of the biosorbent. This variation of the biosorption capacities as a function of contact time is typical for the retention of metal ions by biosorption and has been reported by many studies from the literature which use alginate-based biosorbents [29,30].

For the kinetic modelling of the experimental data, three kinetic models were selected, namely: the pseudo-first order kinetic model, pseudo-second order kinetic model and intra-particle diffusion model. If the first two kinetic models are useful for the establishment of the nature of the elementary steps (physical or chemical) and the number of activity sites involved in the studied biosorption processes [31,32], the third kinetic model will provide a complete description of the elementary diffusion processes that take place [33].

The mathematical equations of each kinetic model and the kinetic parameters calculated for the biosorption of each metal ion on alginate are summarized in Table 1, while the comparison of experimental and theoretical kinetic curves is presented in Figure 3.

It can be observed from Table 1 that the pseudo-second order kinetic model best describes the experimental data obtained at the biosorption of Cu(II), Co(II) and Zn(II) ions on alginate, since the values of the regression coefficients (R^2^) are higher than 0.999 and the biosorption capacities calculated from this model (q^calc^_e_, mg/g) are very close to the experimental ones (q^exp^_e_, mg/g), for all the studied metal ions. Therefore, the retention of Cu(II), Co(II) and Zn(II) ions on alginate was achieved through chemical interactions between metal ions from aqueous solution and superficial functional groups of biosorbent, and these interactions involved two binding sites [34]. Most likely, during the biosorption processes, the replacement of Ca(II) ions from alginate structure with metal ions (Cu(II), Co(II) and Zn(II) ions) from the solution took place, according to Equation (5). This ion exchange equilibrium depended on the affinity of metal ions from solution for the superficial functional groups of alginate, and according to kinetic data (see Table 1), followed the order: Cu(II) > Zn(II) > Co(II).

**Figure 4:**
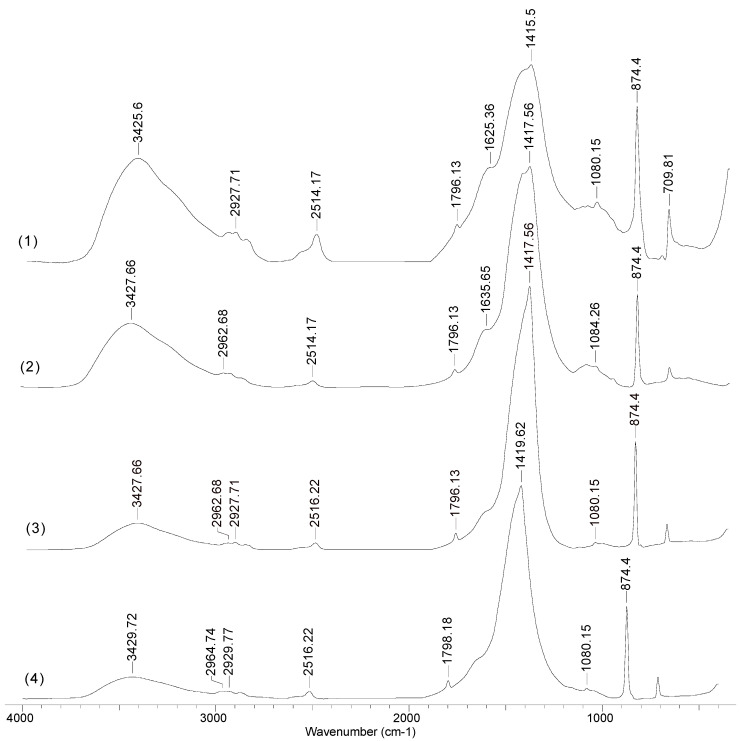
FTIR spectra of alginate, before (**1**) and after (**2**) Cu(II), (**3**) Co(II) and (**4**) Zn(II) ions biosorption, in optimal experimental conditions.

In order to highlight the importance of the ion exchange equilibriums in the studied biosorption processes, the FTIR spectra for alginate were recorded, before and after the retention of metal ions (Figure 4).

The lack of new absorption bands and the insignificant displacements of the maximum wave numbers after metal ions biosorption (spectrum 2–4) clearly show that the biosorption process took place predominantly by ion exchange, and that after metal ion retention, the chemical vicinity of all functional groups (hydroxyl (3425 cm^−1^), carbonyl and carboxyl (1796 and 1625 cm^−1^), ether and esters (1415 cm^−1^) did not change. Therefore, during biosorption, the metal ions (Cu(II), Co(II) and Zn(II)) must reach close to the surface of the alginate particles, where the ion-exchange equilibrium takes place.

Under these conditions, the diffusion of metal ions to and inside alginate particles will play an important role in defining the efficiency of biosorption processes. To evaluate the contribution of elementary diffusion processes to the retention of Cu(II), Co(II) and Zn(II) ions on alginate, the experimental data were modelled using the intra-particle diffusion model. As can be seen from Figure 3 and Table 1, the intra-particle diffusion model describes the experimental data quite well, because the regression coefficients (R^2^) are higher than 0.86, in all cases.

**Figure 5:**
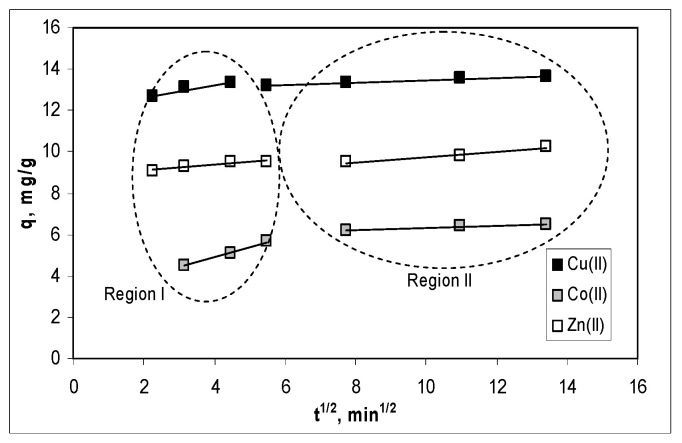
Linear representations of the intra-particle diffusion model for the biosorption of Cu(II), Co(II) and Zn(II) ions on alginate.

However, the linear representations of this model (Figure 5), indicate that the elementary diffusion processes were not the rate limiting steps in the studied biosorption processes. This is because all linear representations (q_t_ vs. t^1/2^), do not pass through the origin [33] and can be clearly delimited into two regions.

According to the theoretical assumptions of this model [33], the first region corresponds to the film diffusion, while the second region characterizes the diffusion of metal ions into the pores of alginate. Comparing the values of the rate constants presented in Table 1, it can be observed that the film diffusion (Region I) is a much more rapid process than the diffusion of metal ions into the pores of the biosorbent (Region II), for all metal ions. In addition, the calculated c_I_ and c_II_ values, which are an indication of the thickness of boundary layer, increase in the order: Cu(II) > Zn(II) > Co(II). All these observations suggest that, in the biosorption processes of Cu(II), Co(II) and Zn(II) ions are mainly involved in the functional groups located on the alginate surface, and that Cu(II) ions have the highest affinity for them, because its concentration in the thickness of boundary layer is the highest.

**Figure 6:**
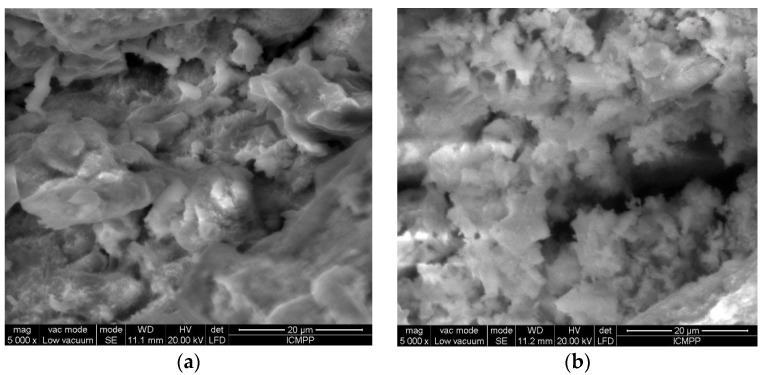

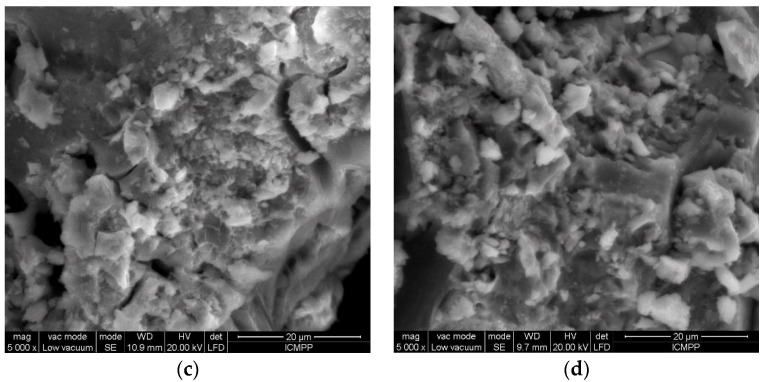
SEM images of alginate before (**a**) and after (**b**) Cu(II), (**c**) Co(II) and (**d**) Zn(II) ion biosorption.

Scanning electron microscope images (SEM) for alginate before and after Cu(II) ion biosorption (Figure 6) were recorded to highlight the surface morphology of biosorbent. As can be observed from Figure 6a, the surface of alginate is irregular, heterogeneous and has pores of different sizes.

After Cu(II) ion biosorption (Figure 6b), the morphology of biosorbent surface seems to be changed, becoming even more heterogeneous and irregular, which indicates that the diffusion of metal ions from the bulk solution and then their binding to the alginate surface was successfully carried out.

### 3.3. Effect of Initial Metal Ions Concentration and Isotherm Modelling

**Figure 7:**
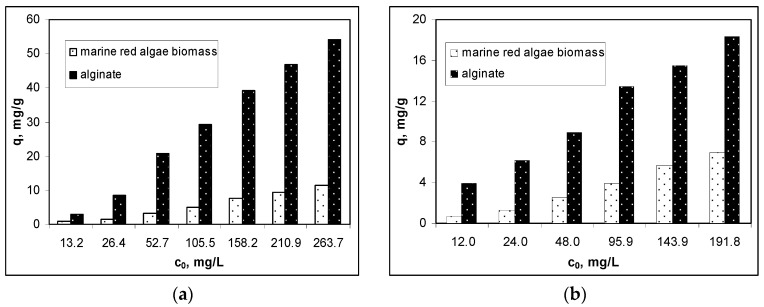

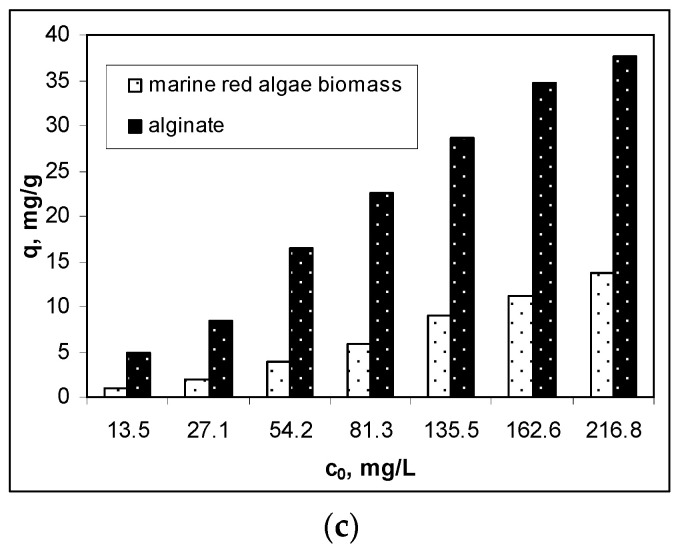
Comparison of the performances of marine red algae biomass and alginate for the biosorption of: (**a**) Cu(II), (**b**) Co(II) and (**c**) Zn(II) ions.

The extraction of alginate from marine red algae biomass was performed in order to obtain a biosorbent with higher performance for removing metal ions from an aqueous solution. In Figure 7, the biosorptive performances of marine red algae biomass and alginate at different initial concentrations of Cu(II), Co(II) and Zn(II) ions, under optimal experimental conditions are presented comparatively.

The experimental results presented in Figure 7 highlight two important aspects, namely: (i) the biosorption capacities of alginate were significantly higher than those obtained for marine red algae biomass, for all studied metal ions and for the entire concentration range and (ii) these differences were higher as the initial concentration of the metal ion was lower. Thus, in the case of Cu(II) ion biosorption, the biosorption capacity of alginate was almost four times higher than the biosorption capacity of marine red algae biomass, throughout the entire concentration range.

In the case of Co(II) ions biosorption, the biosorption capacity of alginate was over six times higher than the biosorption capacity of marine red algae biomass at the lowest initial concentration (12 mg/L), when at the highest initial concentration (192 mg/L), the biosorption capacity of alginate increased 3.21-fold. In the case of Zn(II) ion biosorption, the ratio between the biosorption capacities of alginate and marine red algae biomass varied from 4.75 (at the lowest initial concentration of 13.5 mg/L) to 3.35 (at the highest initial concentration of 216.8 mg/L). These encouraging results, in addition to proving that alginate is one of the main compounds responsible for retaining metal ions on marine algae biomass, as is mentioned in the literature [27,29,30], are a solid argument for its extraction and subsequent use as a biosorbent.

Three isotherm models, namely the Langmuir, Freundlich and Temkin models, have been used to analyze the equilibrium experimental data obtained from the biosorption of Cu(II), Co(II) and Zn(II) ions on alginate, in the mentioned experimental conditions. The selection of the three models was made taking into account that the Langmuir and Freundlich models can be useful for determining the mode (mono- or multi- layer) in which the studied biosorption processes take place [35,36], while the Temkin model will provide information about the nature (physical or chemical) of these processes [37].

**Figure 8:**
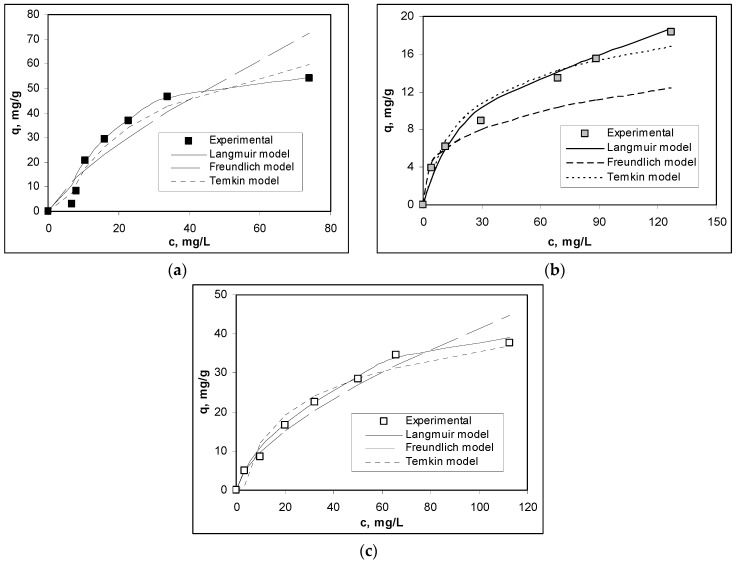
Experimental and theoretical isotherms obtained for the biosorption of (**a**) Cu(II) ions, (**b**) Co(II) ions and (**c**) Zn(II) ions on alginate.(Experimental conditions: pH = 4.4; biosorbent dosage = 2.0 g/L; temperature = 22 °C).

The mathematical equations of these isotherm models are presented in Table 2, together with the values of the characteristic parameters for each metal ion. Moreover, a comparison of the experimental isotherms and those calculated using the mentioned isotherm models is presented in Figure 8.

The values of regression coefficients (*R*^2^) higher than 0.98 for the all studied metal ions (Table 2) suggest that the equilibrium experimental data obtained for the biosorption of Cu(II), Co(II) and Zn(II) ions on alginate are better described by the Langmuir model, compared to the Freundlich and Temkin models (Figure 8). Therefore, the retention of metal ions by alginate occurs at the surface of biosorbent and includes the formation of monolayer coverage. The maximum biosorption capacity (q_max_, mg/g), corresponding to the formation of the monolayer coverage follows the order: Cu(II) > Zn(II) > Co(II) (Table 2), and shows once again that Cu(II) ions have the highest affinity for interactions with superficial functional groups of alginate. On the other hand, the close values of the Langmuir constants (K_L_, L/g) obtained for the three metal ions indicate that in the studied biosorption processes, the same type of interactions are involved (most likely ion exchange).

Table 3 compares the values of maximum biosorption capacity of alginate with other marine algae biomass used as biosorbents in the literature, for retaining Cu(II), Co(II) and Zn(II) ions from aqueous media, under similar experimental conditions. The biosorption capacity of alginate for Cu(II), Co(II) and Zn(II) ions was higher compared with the values obtained for different marine algae biomass (Table 3), which highlights its potential to be used as a biosorbent for removing of metal ions from aqueous media.

Although the Freundlich and Temkin isotherm models do not describe so well the equilibrium experimental data of Cu(II), Co(II) and Zn(II) ions biosorption on alginate (Table 2), the analysis of their characteristic parameters shows that: (i) the n values from the Freundlich equation are in the range of *n* > 1, indicating that the biosorption of the studied metal ions on alginate is a favorable process, and (ii) the low B values from the Temkin isotherm model (which characterize the heat of the biosorption process) allow us to say that the retention of metal ions on alginate involves electrostatic interactions, probably of ion-exchange type, which do not involve the breaking of chemical bonds or the formation of new ones.

### 3.4. Desorption of Metal Ions

Desorption studies are useful in assessing the practical applicability of the studied biosorption processes, as they provide a quantitative characterization of metal ion recovery and the reuse of biosorbents [38,42]. In this study, three biosorption/desorption cycles have been performed using the same sample of alginate, for each metal ion, in a batch system. As a desorption agent, 10 mL of 10^−1^ N HNO_2_ solution which was mixed with 0.2 g of alginate for 4 h of contact time and at room temperature was used. After each desorption step, the alginate was washed with distilled water, treated with 10 mL of 10^−1^ M CaCl_2_ solution for regeneration, and used in another biosorption cycle.

More than 97% of the retained metal ions (98.12% for Cu(II), 97.05% for Co(II) and 97.85% for Zn(II)) were desorbed in the first cycle on the alginate, which means that the recovery of these metal ions is quantitative. In addition, after three biosorption/desorption cycles, the biosorption capacity of this biosorbent was not significantly reduced (4.15% for Cu(II), 5.67% for Co(II) and 4.32% for Zn(II)), which indicates that the treatment of exhausted alginate with 0.1 N HMO_3_ solution does not destroy the superficial structure of the biosorbent, and therefore the alginate can be used repeatedly for the removal of metal ions from aqueous effluents.

## 4. Conclusions

In this study, alginate extracted from marine red algae biomass (*Callithamnion corymbosum* sp.) in alkaline media, was used as biosorbent for the removal of Cu(II), Co(II) and Zn(II) ions from aqueous solution. Batch experiments have shown that the maximum efficiency of the studied biosorption processes is obtained in the following conditions: initial solution pH of 4.4, biosorbent dosage of 2.0 g/L and an ambient temperature (22 °C ± 1 °C). Under these conditions, the biosorption capacity of alginate is more than three times higher than the biosorption capacity of marine red algae biomass for all the studied metal ions. The modeling of the experimental data has shown that the Langmuir isotherm model and pseudo-second kinetic model describe well the biosorption processes of the studied metal ions. The absence of new absorption bands and the insignificant displacements of the maximum wave numbers after metal ion biosorption clearly indicate that the retention of metal ions on alginate occurs predominantly by ion exchange interactions. In addition to the very short time required to reach equilibrium (10 min), the maximum biosorption capacities (q_max_, mg/g) follow the order: Cu(II) (64.52 mg/g) > Zn(II) (37.04 mg/g) > Co(II) (18.79 mg/g), and are higher comparatively than the values obtained for different marine algae biomass. Furthermore, the extracted alginate was used in three successive biosorption/desorption cycles, and the obtained results indicate that all metal ions can be quantitatively recovered at the treatment of exhausted biosorbent with 10^−1^ N HNO_3_ solution, and that after this treatment the structure of alginate does not degrade, which allows its repeated use to remove metal ions from aqueous effluents. All these characteristics highlight that the alginate extracted from marine algae has promising applications in the decontamination of industrial effluent containing metal ions.

## Figures and Tables

**Table 1:** Kinetic parameters calculated for the biosorption of Cu(II), Co(II) and Zn(II) ions on alginate.

Kinetic Model	Mathematical Equation	Parameters	Cu(II)	Co(II)	Zn(II)
Pseudo-first order model	$\log(q_{e}-q_{t})=\log q_{e}-k_{1}\cdot t$	*q*_e_^exp^, mg/g	13.62	6.46	10.24
		*R* ^2^	0.8901	0.9399	0.8375
		*q*_e_^calc^, mg/g	0.71	2.91	1.01
		*k*_1_, 1/min	0.0086	0.0176	0.0028
Pseudo-second order model	$\frac{t}{q_{t}}=\frac{1}{k_{2}\cdot q_{e}^{2}}+\frac{t}{q_{e}}$	*R* ^2^	0.9999	0.9999	0.9999
		*q*_e_^calc^, mg/g	13.64	6.67	10.19
		*k*_2_, g/mg min	0.0107	0.0291	0.0488
Intra-particle diffusion model	$q_{t}=k_{diff}\cdot t^{1/2}+c$	*R* ^2^_I_	0.9208	0.8695	0.9135
		*c*_I_, mg/L	12.06	2.97	8.84
		*k*_diff, I_, mg/g min^1/2^	0.2939	0.4853	0.1318
		*R* ^2^_II_	0.9074	0.9391	0.9510
		*c*_II_, mg/L	12.93	5.85	8.47
		*k*_diff, II_, mg/g min^1/2^	0.0533	0.0475	0.1281

Notations: *q_e_*, *q_t_*—biosorption capacity at equilibrium and at time *t*; *k*_1_—rate constant of pseudo-first order kinetics equation; *k*_2_—pseudo-second order rate constant; *k_diff_*—intra-particle diffusion rate constant, *c*—concentration of metal ions from solution at equilibrium; I and II—region I and II from intra-particle diffusion model linear representations.

**Table 2:** Isotherm parameters calculated for the biosorption of Cu(II), Co(II) and Zn(II) ions on alginate.

Isotherm Model	Mathematical Equation	Parameters	Cu(II)	Co(II)	Zn(II)
Langmuir model	$\frac{1}{q}=\frac{1}{q_{\max}\cdot K_{L}}\cdot \frac{1}{c}$	*R* ^2^	0.9858	0.9895	0.9837
		*q*_max_, mg/g	64.52	18.79	37.04
		*K*_L_, L/g	0.0409	0.0410	0.0424
Freundlich model	$\log q=\log K_{F}+\frac{1}{n}\cdot \log c$	*R* ^2^	0.7893	0.8955	0.9187
		*n*	2.34	3.23	2.59
		*K*_F_, (mg/g)/(L/mg)^1/n^	2.9194	2.8132	2.2977
Temkin model	$q=B\ln A_{T}+B\ln c$	*R* ^2^	0.9531	0.9479	0.9497
		*A*_T_, L/g	0.2099	0.4519	0.3252
		*B*, J/mol	21.75	14.13	20.24

Notations: *q*—biosorption capacity at equilibrium, *q_max_*—maximum biosorption capacity, *K_L_*—Langmuir constant; c-equilibrium concentration of metal ions in solution; *K_F_*—Freundlich constant, *n*—heterogeneity factor; A_T_—Temkin isotherm equilibrium binding constant; B—constant correlated with the heat of biosorption process.

**Table 3:** Comparison of the maximum biosorption capacity of alginate for studied metal ions with different marine algae biomass.

Marine Algae Biomass	pH/Biosorbent Dosage	Cu(II)	Co(II)	Zn(II)	Reference
*Ulva fasciata* sp.	5.0/3.3 g/L	26.88	-	13.50	[38]
*Laminaria* sp.	4.3/3.0 g/L	61.59	-	54.26	[39]
*Padina sanctae crucis* sp.	6.0/4.0 g/L	13.99	13.73	-	[40]
*Codium vermilara* sp.	5.0/0.5 g/L	16.90	-	23.80	[41]
*Callithamnion corymbosum* sp.	4.4/2.0 g/L	24.25	9.89	19.12	This study
Alginate	4.4/2.0 g/L	64.52	18.79	37.04	This study
